# Is maximal oxygen consumption an appropriate metric for metabolic health?

**DOI:** 10.1007/s00421-025-05875-2

**Published:** 2025-07-03

**Authors:** Dale I Lovell, Max Stuelcken, Alexander Eagles

**Affiliations:** 1 School of Health, The University of the Sunshine Coast, Maroochydore, QLD 4556 Australia; 2 Timeless Medical Australia, Maroochydore, QLD 4556 Australia

**Keywords:** Metabolic flexibility, Maximal oxygen consumption, Submaximal exercise

## Abstract

Cardiorespiratory fitness (CRF), assessed by maximal oxygen consumption ($\dot{\text{V}}$O_2_ max) testing, is a strong predictor of chronic disease and all-cause mortality. However, recent evidence suggests that $\dot{\text{V}}$O_2_ max may lack specificity and sensitivity in assessing metabolic health, particularly mitochondrial function, which is associated with metabolic diseases such as type 2 diabetes, insulin resistance, and metabolic syndrome. While aerobic training leads to improvements in mitochondrial function, studies have found a disparity between $\dot{\text{V}}$O_2_ max and mitochondrial content, with some individuals showing increases in mitochondrial oxidative capacity without changes in $\dot{\text{V}}$O_2_ max. Furthermore, the criteria used to determine $\dot{\text{V}}$O_2_ max, such as the plateau in oxygen consumption, may not be achieved by all individuals, leading to inaccurate assessments. Technological advances in metabolomics and lipidomics may provide insights into metabolic health, but their cost and practicality for routine use in clinical settings remain a challenge. Alternatively, indirect calorimetry during submaximal exercise has shown promise as a non-invasive marker of mitochondrial function and metabolic flexibility. However, further research is needed to establish appropriate protocols and analyses for various populations.

## Introduction

It is well established that a low level of cardiorespiratory fitness (CRF) is a strong predictor of chronic disease and all-cause mortality (Ross et al. 2016). The maximal oxygen consumption ($\dot{\text{V}}$O_2_ max) test is considered the gold standard measure of CRF and is frequently utilized to evaluate metabolic health (Ross et al. 2016). Metabolic health refers to the optimal functioning of the body's metabolic processes, including blood sugar regulation, cholesterol levels, blood pressure, and body fat distribution (Araujo et al. 2019). However, there is a growing body of evidence indicating that a $\dot{\text{V}}$O_2_max test may lack specificity and validity as an appropriate metric for the assessment of metabolic health (Brun et al. 2022; Deboeck et al. 2023; San-Millan 2023). An important contributor to metabolic health is mitochondrial function, which is associated with various metabolic diseases such as type 2 diabetes, insulin resistance and metabolic syndrome (MetS) (Mancilla et al. 2023; San-Millan 2023). Therefore, the assessment of mitochondrial function is essential for the detection of metabolic disease and validation of the effects of interventions aimed at improving metabolic health.

## Components of maximal oxygen uptake

Multifactorial modelling based on oxygen conductance has demonstrated that the attainment of $\dot{\text{V}}$O_2_ max is interconnected through a series of components, including pulmonary gas exchange, cardiac output, blood oxygen delivery, and oxygen diffusion into the muscle (Ferretti 2014; Wagner 2023). Each of these components can independently influence $\dot{\text{V}}$O_2_ max, and any variation in one of these components may lead to alterations in the overall oxygen delivery from the atmosphere to the mitochondria (di Prampero 1992; Wagner 2023). As a result of the integrated influence of the various components involved in achieving $\dot{\text{V}}$O_2_ max, it is not feasible to ascertain whether the capacity of the mitochondria is being evaluated. This may explain why some studies have found a significant correlation between the mitochondria and $\dot{\text{V}}$O_2_ max (Granata et al. 2018; Holloszy 1967), while others have found considerable disparity between $\dot{\text{V}}$O_2_ max and mitochondrial content (Boushel et al. 2011; Granata et al. 2016; Jacques et al. 2021; Lundby & Jacobs 2016; Montero et al. 2015; Zhang et al. 2021). Additionally, Venckunas and colleagues (Venckunas et al. 2024) recently reported that muscle mitochondrial power estimated from near-infrared spectroscopy was not correlated with whole-body aerobic capacity and that different factors may underpin the two indices of aerobic capacity. Taken together, these studies highlight the lack of specificity and sensitivity of the $\dot{\text{V}}$O_2_ max test for assessing metabolic health.

## $\dot{\mathbf{V}}$O_2_ max criteria

Further confounding the relationship between the assessment of metabolic health and $\dot{\text{V}}$O_2_ max is the criterion used to determine whether an individual reaches their maximal aerobic capacity. The initial criterion for attaining $\dot{\text{V}}$O_2_ max developed by Hill and Lupton in the early twentieth century was based on a plateau in oxygen consumption occurring with increasing workloads (Niemeyer et al. 2021). However few participants who undergo a $\dot{\text{V}}$O_2_ max test actually achieve a plateau. In particular, sedentary participants and clinical populations may stop exercising before their $\dot{\text{V}}$O_2_ max is reached (Poole & Jones 2017). This was demonstrated by Moreno-Cabanas and colleagues, who investigated the accuracy of a graded exercise test (GXT) to assess improvements in $\dot{\text{V}}$O_2_ max in unfit individuals with metabolic syndrome (Moreno-Cabañas et al. 2020a).Using the plateau criterion, they reported that the GXT overestimated $\dot{\text{V}}$O_2_ max improvements by 41% and underestimated $\dot{\text{V}}$O_2_ max improvements in 59% of the participants. The inability to accurately identify $\dot{\text{V}}$O_2_ max in metabolically unhealthy individuals may help to explain the findings of recent studies that reported no difference in aerobic capacity in individuals with or without clinically diagnosed metabolic syndrome (Deboeck et al. 2023; Petersen et al. 2024). To overcome the limitations of the plateau criterion, other additional methods have been employed, such as the use of a secondary criterion achieved during a $\dot{\text{V}}$O_2_ max test as well as the implementation of a verification phase. Although the secondary criterion has been criticized as an inaccurate method of $\dot{\text{V}}$O_2_ max estimation (Poole & Jones 2017), the verification phase has been shown to have some merit, particularly for obese metabolic individuals with low fitness levels (Moreno-Cabañas et al. 2020a, b).

## Defining metabolic syndrome and metabolic health

Over the last three decades, there has been considerable debate over the exact definition of MetS (Neeland et al. 2024). In 2009, several international organisations released a statement harmonizing the criteria for MetS to include raised blood pressure, dyslipidaemia (raised triglycerides and lowered high-density lipoprotein cholesterol), raised fasting glucose, and central obesity (Alberti et al. 2009). Three abnormal findings out of 5 would indicate a person has MetS. In most countries, the prevalence of MetS is increasing with over 40% of the population meeting the criteria (Neeland et al. 2024). MetS is a chronic and progressive pathophysiological state that can take many years to develop, with early identification and intervention being the key to reducing the health effects of MetS. With this in mind, a recent study proposed the concept of optimal metabolic health as a means to identify those at risk before the appearance of MetS symptoms (Araujo et al. 2019). Metabolic health is defined as having optimal levels of five factors: blood glucose, triglycerides, high-density lipoprotein cholesterol, blood pressure and waist circumference, without the need for medications (Araujo et al. 2019). Most alarmingly, only 12% of the US population were identified as metabolically healthy (Araujo et al. 2019). However, neither of the two previously mentioned approaches assesses mitochondrial function, which has been identified as a key component of metabolic health (San-Millan 2023; Shoemaker et al. 2023). Mitochondrial dysfunction has been strongly linked to insulin resistance, obesity and MetS (Muoio 2014; Sangwung et al. 2020). The difficulty in assessing mitochondrial function, in particular, within a large population setting, may be a cause for its omission.

## Mitochondrial function and metabolic flexibility

Metabolic flexibility (MF) is defined as the ability of an individual to maintain energy homeostasis by matching fuel availability with metabolic demand (Lovell et al. 2025; San-Millan & Brooks 2018; Shoemaker et al. 2023). Central to MF is mitochondrial function and there is strong evidence supporting the assessment of MF as a method of determining mitochondrial health and as a possible indicator of future chronic disease (Brun et al. 2022; Galgani & Fernandez-Verdejo 2021; San-Millan 2023; Shoemaker et al. 2023). Furthermore, it has been reported that metabolic inflexibility precedes glucose intolerance following a period of bed rest (Rudwill et al. 2018). A test of MF must be able to assess the capacity of skeletal muscle to adjust its utilization of substrate pathways in response to a metabolic challenge. Current methods include the assessment of oxidative enzymes from muscle biopsies and/or the use of cell cultures, both of which are invasive and not practical for the general population (Mancilla et al. 2023). Non-invasive methods include the use of nuclear magnetic resonance and magnetic resonance spectroscopy, but this technology is costly and not feasible for routine use. Alternatively, a simple method for indirectly measuring mitochondrial function during exercise was proposed that can be performed on a large scale in an ambulatory manner (San-Millan & Brooks 2018).

## Sub-maximal exercise and the assessment of metabolic flexibility

Exercise provides an ideal challenge to the metabolic environment of skeletal muscle because fat and carbohydrate (CHO) use within the mitochondria change depending on the exercise intensity placed on the body. This concept is similar to a cardiology stress test, where the cardiovascular system is placed under stress for the early detection of cardiovascular disease and abnormalities not necessarily observed at rest (Gevaert et al. 2023). The most common method of assessing the metabolic response to exercise is through indirect calorimetry (Amaro-Gahete et al. 2019). Fat and CHO oxidation are calculated using a stoichiometric equation to determine maximal fat oxidation (MFO) and the exercise intensity eliciting MFO (Fat_max_) (Frayn 1983). A greater reliance on CHO oxidation or conversely low MFO values at low-moderate exercise-intensities indicates poor MF. One of the first studies to examine MFO and Fat_max_ during 4–6 sub-maximal exercise intensities found Fat_max_ values occurred at approximately 56% of $\dot{\text{V}}$O_2_ max in moderately trained cyclists (Achten et al. 2002). However, there was considerable variation in Fat_max_ values within the group (55–72% of $\dot{\text{V}}$O_2_ max). Since this early study many others have investigated ways to assess MFO and Fat_max_ in a variety of population groups while using differing protocols (Amaro-Gahete et al. 2019; Brun et al. 2022; Maunder et al. 2018; San-Millan & Brooks 2018). As a result, Fat_max_ values reported in the literature range from as low as 20% up to 80% of $\dot{\text{V}}$O_2_ max. Recently the measurement of blood lactate levels was incorporated during a sub-maximal exercise test to assist with the identification of Fat_max_ values (San-Millan & Brooks 2018). Blood lactate was found to be negatively correlated with fat oxidation and provided additional support for the notion that this test can indirectly measure MF and mitochondrial function. Furthermore, a recent comparison between invasive and non-invasive markers of mitochondrial function found exercise efficiency (calculated with indirect calorimetry) was the best non-invasive marker of mitochondrial respiratory capacity (Mancilla et al. 2023).

## Advantages/disadvantages of sub-maximal exercise testing

There are several important advantages of sub-maximal testing for MFO and Fat_max_. Firstly, the low to moderate intensity used for the sub-maximal exercise test, overcomes the potential confounding influence of the components involved in attaining a $\dot{\text{V}}$O_2_ max mentioned earlier in this paper. Secondly, it avoids the safety concerns for the elderly and other at-risk clinical populations during a maximal exercise test (Beltz et al. 2016). Thirdly, it does not require high levels of motivation and maximal effort which is often associated with considerable pain and discomfort for the participant. Finally, the timing of the sub-maximal test is not as important as it is for a $\dot{\text{V}}$O_2_ max test. For example, if testing an athlete, the timing of the $\dot{\text{V}}$O_2_ max test needs to be carefully considered so as not to impair performance on subsequent days (Coquart et al. 2014).

However, certain concerns must be addressed prior to employing the sub-maximal exercise test for the precise determination of substrate utilization and mitochondrial function. Metabolic responses may vary significantly among individuals based on factors such as sex, age, training status, body composition, and various clinical conditions. Furthermore, variations in methodological approaches, such as protocol design, exercise modality, and the selection of stoichiometric equations, may exacerbate the complexity of the issue. These challenges can be partially mitigated by grouping participants with similar characteristics and implementing more consistent protocols (Lovell et al. 2025).

## Conclusion

Recent research has acknowledged the necessity for an improved understanding of the non-invasive assessment of substrate utilization and regulation during exercise (Brun et al. 2022; San-Millan 2023). This knowledge would facilitate not only the improvement of exercise performance but also enable direct assessment of metabolic and mitochondrial health. An individual's $\dot{\text{V}}$O_2_ max is frequently utilized as a proxy measure of metabolic health. However, a $\dot{\text{V}}$O_2_ max test is a broad measure of the body's cardiovascular and metabolic systems and may lack the required precision and specificity for metabolic and mitochondrial health assessments. Emerging evidence suggests that indirect calorimetry assessed during a submaximal exercise test may serve as a suitable alternative to the traditional $\dot{\text{V}}$O_2_ max test for the assessment of mitochondrial and metabolic health. Whilst, there are some methodological issues surrounding the use of submaximal exercise and indirect calorimetry that need to be addressed before this test becomes an acceptable assessment of mitochondrial health (Amaro-Gahete & Ruiz 2018; Lovell et al. 2025), it provides considerable promise as an easy, time-efficient, and relatively non-invasive measure of MF and mitochondrial health.

## Data Availability

Data sharing is not applicable to this article as no new data were created or analysed in this study.
